# Pseudotype-Based Neutralization Assays for Influenza: A Systematic Analysis

**DOI:** 10.3389/fimmu.2015.00161

**Published:** 2015-04-29

**Authors:** George William Carnell, Francesca Ferrara, Keith Grehan, Craig Peter Thompson, Nigel James Temperton

**Affiliations:** ^1^Viral Pseudotype Unit, Medway School of Pharmacy, The Universities of Greenwich and Kent at Medway, Chatham Maritime, Kent, UK; ^2^Department of Zoology, University of Oxford, Oxford, UK; ^3^The Jenner Institute Laboratories, University of Oxford, Oxford, UK

**Keywords:** influenza, hemagglutinin, pseudotype, neutralization assay, universal vaccine, lentiviral vector, retroviral vector

## Abstract

The use of vaccination against the influenza virus remains the most effective method of mitigating the significant morbidity and mortality caused by this virus. Antibodies elicited by currently licensed influenza vaccines are predominantly hemagglutination-inhibition (HI)-competent antibodies that target the globular head of hemagglutinin (HA) thus inhibiting influenza virus entry into target cells. These antibodies predominantly confer homosubtypic/strain specific protection and only rarely confer heterosubtypic protection. However, recent academia or pharma-led R&D toward the production of a “universal vaccine” has centered on the elicitation of antibodies directed against the stalk of the influenza HA that has been shown to confer broad protection across a range of different subtypes (H1–H16). The accurate and sensitive measurement of antibody responses elicited by these “next-generation” influenza vaccines is, however, hampered by the lack of sensitivity of the traditional influenza serological assays HI, single radial hemolysis, and microneutralization. Assays utilizing pseudotypes, chimeric viruses bearing influenza glycoproteins, have been shown to be highly efficient for the measurement of homosubtypic and heterosubtypic broadly neutralizing antibodies, making them ideal serological tools for the study of cross-protective responses against multiple influenza subtypes with pandemic potential. In this review, we will analyze and compare literature involving the production of influenza pseudotypes with particular emphasis on their use in serum antibody neutralization assays. This will enable us to establish the parameters required for optimization and propose a consensus protocol to be employed for the further deployment of these assays in influenza vaccine immunogenicity studies.

## Influenza Pseudotypes

Influenza is a respiratory syndrome caused by three of six genera in the orthomyxoviridae family, influenza A, B, and C. A putative fourth genus (influenza D) has recently been characterized and proposed (1). Influenza A is the most widespread, its various subtypes are classified according to their antigenically variable surface glycoproteins: hemagglutinin (HA, H1–H18) and neuraminidase (NA, N1–N11). The virion consists of a segmented negative sense genome encapsidated in ribonucleoprotein complexes, which are surrounded by a matrix shell and lipid envelope containing the two surface glycoproteins and the M2 ion channel. Influenza A is the primary source of the human seasonal form of the disease, responsible for up to 500,000 deaths per annum as well as deaths caused by pandemics such as those occurring in 1918, 1957, 1968, and 2009 (2). Consequently, vaccines against influenza need to be regularly updated to match predicted circulating strains that are constantly escaping from vaccine protection through a mechanism known as antigenic drift. Influenza A is primarily associated with wild fowl/birds in the case of the majority of subtypes and can reassort with human strains through antigenic shift to yield human compatible viruses with previously un-encountered surface epitopes. Pigs are usually considered to be the mixing vessel for reassortment as they express a mixture of α-2,3 and α-2,6 sialic acid linkages. Influenza virus research is often hindered by the requirement for expensive biosafety precautions, especially in the case of the highly pathogenic avian influenza (HPAI, e.g. H5N1, H7N1) or pandemic strains.

Pseudotypes or pseudotype particles are chimeric “viruses” consisting of a surrogate virus core surrounded by a lipid envelope with the surface glycoproteins of another virus, such as HA. By removing the genetic element of the virus being studied and replacing it with a suitable reporter, viruses, especially HPAI, can be studied in this safer, single cycle system. The comparative safety of pseudotype viruses circumvents the need for restrictive, expensive, and widely unavailable high-category biosafety facilities, increasing access to research groups interested in highly pathogenic viruses.

This review is a systematic analysis encompassing a wide range of peer-reviewed literature in English concerning the production and use of pseudotypes bearing influenza glycoproteins to date. For the purpose of this review, pseudotypes will be defined as replication-deficient viruses containing a viral core from one species and bearing glycoproteins from another that are not represented in the genome. Literature was gathered by searching for “influenza pseudotypes” using Google Scholar and NCBI PubMed. The resulting list of publications was expanded by following up cited references and finally, those falling outside of our pseudotype definition or not specifically using influenza pseudotypes were excluded from the sections on production, transduction, and neutralization.

This review will be useful to those interested in the production of pseudotypes for use in immunogenicity testing of pre-clinical influenza vaccines, whether in human or animal settings, and including “universal vaccine” candidates. Influenza serological studies such as the measurement of seroprevalence will benefit from this manuscript, which will also help to inform the process of validation of pseudotype-based assays to clinical end-point. Furthermore, studies utilizing chimeric HA proteins in order to differentiate between stalk and head directed antibodies will be discussed.

## Pseudotype Components

### Cores and reporters

The core and its associated genome containing a reporter are the backbone of the pseudotype system, which can be used to study the properties of selected entry proteins. The use of cores from lentiviral human immunodeficiency virus (HIV) and gammaretroviruses such as murine leukemia virus (MLV) predominate in the influenza pseudotype literature. Recent development of systems involving rhabdoviruses, in particular the vesicular stomatitis virus (VSV), has also been used to produce pseudotype cores with promising results (3, 4).

### Retroviral and lentiviral cores and vectors

Retroviral and lentiviral vectors are complex systems, which will be explained in simple terms specific to the production and use of pseudotypes. Pseudotype core and vector systems have been reviewed in detail (5, 6).

The primary genes provided by retroviral and lentiviral systems are *gag* and *pol*. In the case of HIV, *gag* provides the structural proteins p18, p24, and p15, whereas *pol* provides the integrase and reverse transcriptase in conjunction with the p10 protease required for cleavage and maturation of each distinct protein from their respective polypeptide chain (7, 8). Reporter constructs are associated with their respective cores based on the Psi (Ψ) packaging element incorporated in the vector design process, making them specific to the surrogate species used.

Human immunodeficiency virus cores are derived from several different origins between laboratory groups. First generation pNL4-3 vectors are well represented and the pNL4-3-Luc.E-R-variant is the most commonly used (9–14). The pNL4-3.Luc.E-R-replication deficient proviral HIV-1 clone is derived from the pNL precursor but has inhibitory frame shifts in the *env* and *vpr* genes as well as a luciferase reporter gene cloned into *nef* and the entire construct is incorporated into progeny pseudotypes. The vector’s life cycle mimics that of HIV, using the Ψ element to allow encapsidation into nascent pseudotypes and long terminal repeat (LTR) regions bearing the U3 promoter, which with the aid of *tat*, permit the expression of the viral proteins after integration into the host genome. The rev responsive element (RRE) allows nuclear export of viral messenger RNA (mRNA), including the reporter gene transcript, which is the measure of output for this system. Due to the incorporation of the HIV core genes into the same integrated construct as the reporter, transduced cells may possibly produce luciferase containing cores alongside its transcribed enzyme, which could potentially interfere with luciferase activity.

Another commonly used HIV core vector is pCMV ΔR8.2, a relation of pCMV ΔR8.9, which still contains intact *vif*, *vpr*, *vpu*, and *nef* genes (15–20).

A further approach uses the second generation HIV vector p8.91 that also originates from pCMV ΔR8.9 and ΔR9 (15, 21). The p8.91 vector is a modified HIV-1 clone, lacking the Ψ sequence as well as the *env*, *vif*, *nef*, *vpu*, and *vpr* genes and is widely used in the articles studied (22–25). The cytomegalovirus promoter is used in lieu of LTR-based promotion, meaning that p8.91 provides the necessary genes for the production of the core but the proviral and packaging elements (LTRs, RRE, and Ψ) are transferred to a separate plasmid bearing the reporter gene. Thus, the reporter construct will be incorporated into nascent virions and integrated into the transduced cell’s genome, whereupon the LTRs and RRE will act to enhance expression. In the case of the commonly used firefly luciferase or green fluorescent protein (GFP) plasmids pCSFLW or pCSGW, a safety component is incorporated through a deletion in the 3′ LTR (U3 promoter region), creating so called self-inactivating (SIN) vectors (26, 27).

Third generation vectors have also been used. In this instance, HIV structural and accessory genes are separated from *rev*, which is provided in *cis* on an additional plasmid. The third generation Invitrogen ViraPower Lentiviral Expression System was used in several cases using the plasmids pLP1 and pLP2 (28–31).

Murine leukemia virus cores are less widely used but provide similar *gag* and *pol* elements to HIV vectors (32–38). One MLV core used consists of *gag* and *pol* under the effect of a CMV promoter, a vector which has been shared across various laboratories (39–41). In this instance, the vector originates from pCI G3 N, B, or NB, which are differentially restricted in certain murine cells based on the mouse resistant gene alleles Fv1^N^ and Fv1^B^ (42). The reporters used in this system are derived from CLONTECH vectors LNCX and pIRES2-EGFP (39, 41). Another described MLV plasmid, pkatgagpolATG originates from the ecotropic Moloney MLV and strain 4070A (17).

Minor differences have been observed when pseudotyping HIV or MLV cores with influenza glycoproteins (43). Therefore, the question of which core to use to produce pseudotypes is often down to choice, preference, and availability (44).

**Figure 1:**
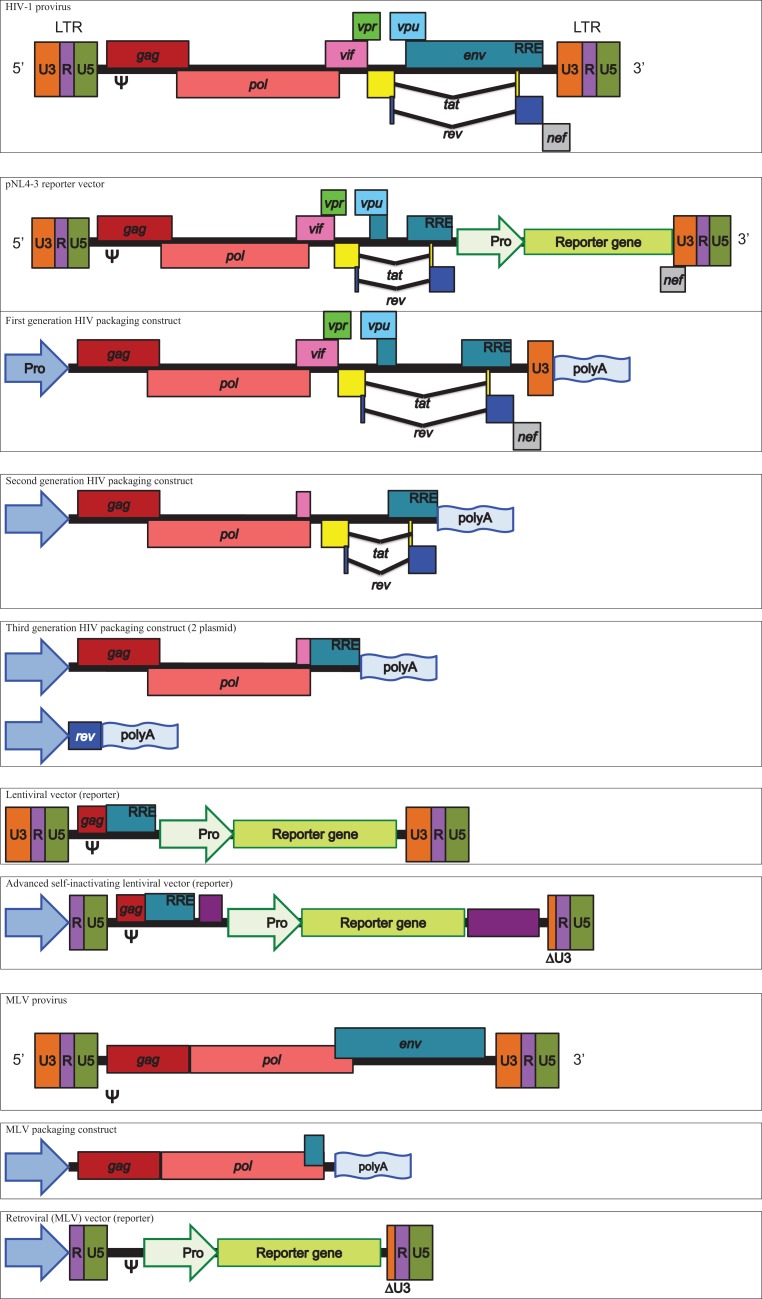
**Schematic representation of HIV and MLV derived packaging constructs and vectors**.

See Figure 1 for schematic representations of packaging constructs and vectors.

### Rhabdoviruses

Recombinant VSV viruses are produced expressing GFP in place of the resident VSV envelope glycoprotein (VSV-G). In certain cases, HA and NA or simply HA are also added to the VSV genome. These additions produce a replication-competent virus, which will promote GFP production in infected cells (4). As these recombinant viruses are not limited to a single cycle of replication, they lack the safety element found within other systems.

A safer VSV-based alternative involves transfection of surface protein encoding plasmids (HA/NA) into cells and subsequent infection with a recombinant VSV. In this way, one can produce VSV pseudotyped with influenza surface proteins, which lack entry-glycoproteins in its resident genome, rendering the second generation of virus infection-incompetent (3).

### Reporter systems

The output of the pseudotype system is based on the incorporated reporter, which mimics the genome of the surrogate virus. In the case of HIV or MLV surrogates, the reporter will often be incorporated into the pseudotype in RNA form, which upon transduction will be reverse transcribed, translocated to the nucleus, and integrated into the host cell genome. The reporter will then be produced by the host cell and can be used to measure transduction efficiency.

The primary reporter used in influenza pseudotypes is firefly derived luciferase (45–53). Relative luminescence units (RLU) or relative luciferase activity (RLA) are used as output, measured by lysing transduced cells and adding substrate for the luciferase enzyme, the signal from which is then read using a luminometer.

Green fluorescent protein is also commonly used, in which case transduction efficiency is determined by counting the number of fluorescing cells via epifluorescence microscopy or fluorescence-activated cell sorter (FACS) (54, 55).

Other reporters such as *lacZ* (29, 54, 56, 57) as well as *Gaussia* (58) and *Renilla* (59, 60) luciferase are also used to a lesser extent.

### Influenza envelope proteins: Hemagglutinin

The trimeric attachment and fusion protein HA is the principal constituent of the influenza virus envelope, alongside NA and M2. Attachment to sialic acid residues on target cell membranes triggers endocytosis and pH-dependent exposure and engagement of the fusion peptide, mediating entry of the virus (61). This process is the basis on which influenza neutralization assays are founded – the exploitation of attachment and entry for the study of HA-directed antibodies and their neutralizing ability. Analysis has permitted classification of influenza A subtypes into two distinct groups: group 1 containing subtypes 1, 2, 5, 6, 8, 9, 11, 12, 13, 16, 17, and 18 and group 2 containing 3, 4, 7, 10, 14, and 15 (62–64). Subtypes within each group are often subdivided into clades with further sequence dissimilarity.

**Figure 2:**
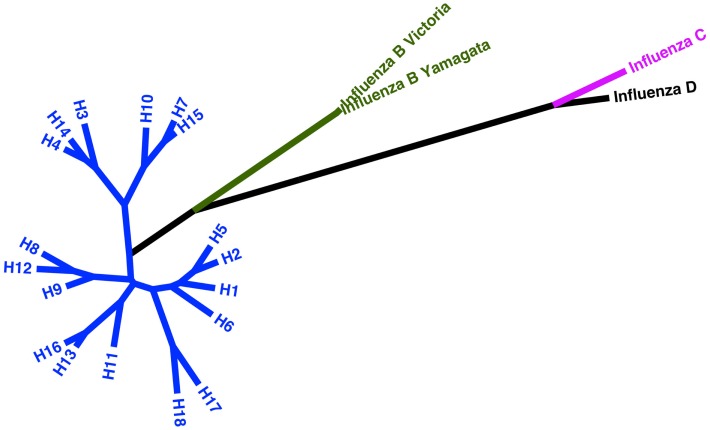
**Phylogeny of current influenza subtypes using the HA glycoprotein**. Maximum likelihood tree representing amino acid sequences of the HA glycoprotein for influenza A, B, and C virus as well as putative influenza D. The tree inferred is based on MUSCLE alignment of downloaded sequences conducted using MEGA 5.2 under the WAG + G model (four categories). The phylogenetic tree with the highest log likelihood (−16773.4044) is shown. The tree is drawn to scale, with branch lengths measured in the number of substitutions per site. The analysis involved 22 amino acid sequences. All positions containing gaps and missing data were eliminated. There were a total of 538 positions in the final dataset (66, 67).

**Figure 3:**
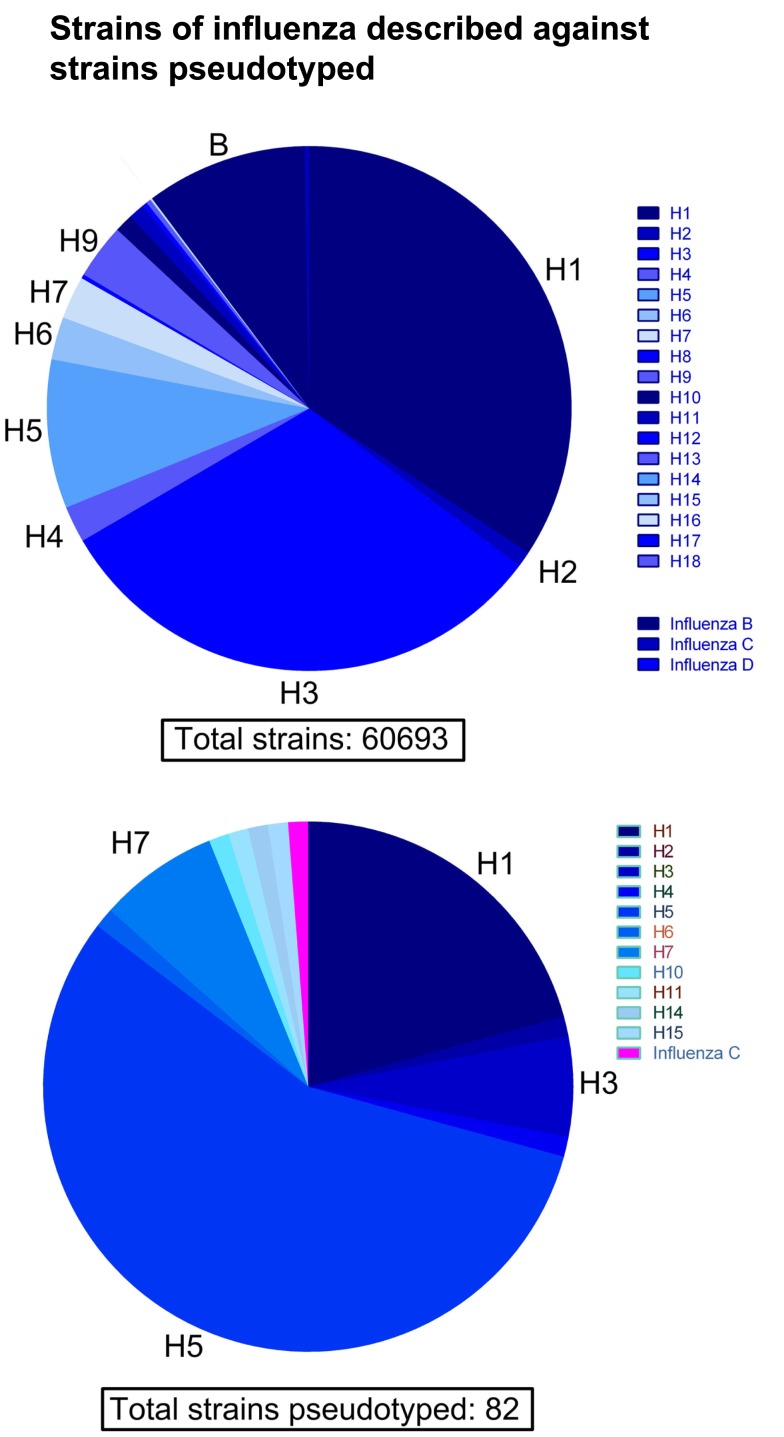
**Comparison of influenza HA sequences described against strains pseudotyped**. Out of a total of 60,693 HA amino acid sequences extracted from NCBI GenBank, the vast majority come from subtypes H1, H3, H5, and influenza B. Conversely, the current number of different subtypes and strains of HA used to produce pseudotypes is 82. The majority of pseudotyped strains come from subtypes H1, H3, and especially H5.

See Figure 2 for a phylogeny of influenza groups and Figure 3 for influenza strains pseudotyped with HA compared to HA sequence entries currently in NCBI GenBank. A wide variety of influenza A strains exist and have been pseudotyped, influenza B is grouped into two distinct lineages (Yamagata and Victoria) and has yet to be pseudotyped. Influenza C pseudotypes have been produced using a VSV core (65).

#### Codon optimization, synthesized genes

Codon optimization has been employed for several commercially synthesized genes, which are sometimes used concurrently with extracted wild type viral sequences depending on availability [Genscript, Gene Art, Integrated DNA technologies (54, 68–73)]. Recursive PCR has been used in some cases to produce the same end product (16, 68, 74, 75). In the context of pseudotype production, codon optimization is performed based on the assumption that conforming to codon-bias within producer cells will increase production of proteins and pseudotype yields.

### Influenza envelope proteins: Neuraminidase

As with wild type influenza virus, NA is required for the exit of influenza pseudotypes via its cleavage of surface sialic acid molecules on producer cells. However, it is common to circumvent the requirement of NA expression for pseudotype production by the treatment of cultured cell lines with commercial exogenous bacterial NA 24 h after transfection (59, 76–78). This 24 h time period requires optimization to allow maximal budding of pseudotypes and minimal loss through transduction of producing cells. Exogenous NA treatment is often used in neutralization studies in order to prevent NA directed antibodies from providing a neutralization signal. However, several studies opt to incorporate an NA plasmid such as that from influenza B/Yamagata/16/88, A/Shanghai/37T/2009, A/Thailand/1(KAN)-1/04, or A/Puerto Rico/8/1934 (16, 58, 79–85).

Several recent articles have characterized sialic acid binding attributes of neuraminidases sharing particular genetic characteristics. New mutations have been characterized such as G147R in the A/WSN/33 strain that has been shown to rescue HA-binding deficient viruses. The G147R mutation is present in a range of strains including representatives of pandemic H1N1 and chicken H5N1 (86, 87).

### Influenza envelope proteins: M2

It is also possible to incorporate the M2 ion channel into influenza pseudotypes in order to study its effect on the production process. However, the M2 role in acidification of the wild type influenza virus core is not required for the dissociation of pseudotype cores as they are derived from non-influenza viruses, which achieve release of their genetic material (i.e., a luciferase reporter gene transcript) through different mechanisms. Therefore, M2 is not required for the production of influenza pseudotypes despite being shown to have an effect on yields and infectivity (88, 89). There are reports of M2 incorporation increasing pseudotype particle yields such as H7 A/FPV/Rostock, and for H1N1 pseudotypes (29, 88). M2 has been shown to influence the budding of wild type influenza and consequently, this may be the mechanism through which M2 expression increases the reported pseudotype yields (90).

### Proteases

As HA is produced and trafficked through the secretory pathway it requires proteolytic cleavage in order to become fusion competent. Proteolytic cleavage is mediated by certain host cell proteases and restricts certain subtypes to epithelial cells where these required proteases are expressed. While this is achieved naturally in wild type infection, a cleavage component must be incorporated into pseudotype production workflows in order to achieve optimal yields. This is because in producer cell lines the required proteases are either not expressed or are expressed, but not at sufficient levels to make the pseudotypes fusion competent.

In order to mimic the proteolytic properties of the natural host cells of influenza, protease encoding plasmids can be transfected alongside the other requisite plasmids in order to induce transient expression within the same timeframe as the production of pseudotypes. The serine transmembrane protease (TMPRSS2) and the human airway trypsin (HAT), which cleave wild type influenza (91) have been used successfully in several studies for pseudotype production (17, 28, 70, 78, 92–96). TMPRSS4, another serine protease has also been used to successfully cleave wild type and influenza lentiviral pseudotypes (97).

However, the addition of a protease encoding plasmid can be side-stepped through cleavage post-production using tosyl phenylalanyl chloromethyl ketone (TPCK) treated trypsin (17, 28, 92, 98). TPCK inhibits the less specific proteolytic elements of chymotrypsin, restricting the treatment process to the cleavage of peptide bonds required for HA maturation (99, 100).

TPCK-trypsin concentrations used for the production of pseudotypes generally ranged from 1 to 50 μg/ml. However, one study reported increased transduction when used at concentrations above 40 μg/ml for H1N1 pseudotypes (101). Incubation ranged from 10 min at room temperature to the more usual 1 h at 37°C. TPCK-trypsin treatment is typically carried out an hour before transduction. The enzyme is then neutralized before transduction using commercial trypsin inhibitors, in some cases originating from soybean (28, 92).

HA derived from HPAI strains that contain a polybasic cleavage sequence in the HA0 protein are cleaved by a wider range of proteases that are ubiquitous in cells. This allows the omission of protease plasmids or TPCK-trypsin treatment in HPAI pseudotype production (102). In some cases, the polybasic cleavage site of HPAI strains have been integrated into other HAs in an attempt to produce pseudotypes without the protease plasmid requirement, or to give strains similar entry characteristics (60, 73).

**Figure 4:**
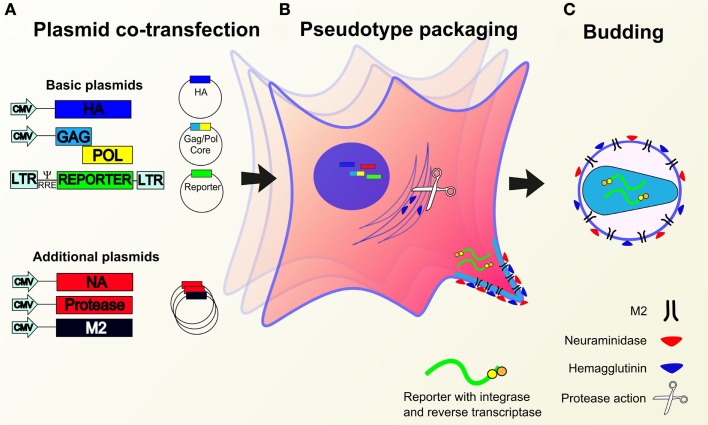
**Production of lentiviral or retroviral pseudotypes**. **(A)** Essential (containing HA, packaging construct *gag pol*, reporter construct) and/or additional (NA, protease, M2) expression plasmids are co-transfected into HEK293T producer cells. **(B)** Plasmids migrate to the nucleus whereupon genes are expressed leading to the production of pseudotype proteins and the reporter RNA construct. Cleavage of HA is mediated by transfected or cellular proteases. **(C)** Pseudotype proteins are packaged by the cell and budding occurs at the cell membrane to yield pseudotypes bearing desired glycoproteins and incorporated reporter.

**Figure 5:**
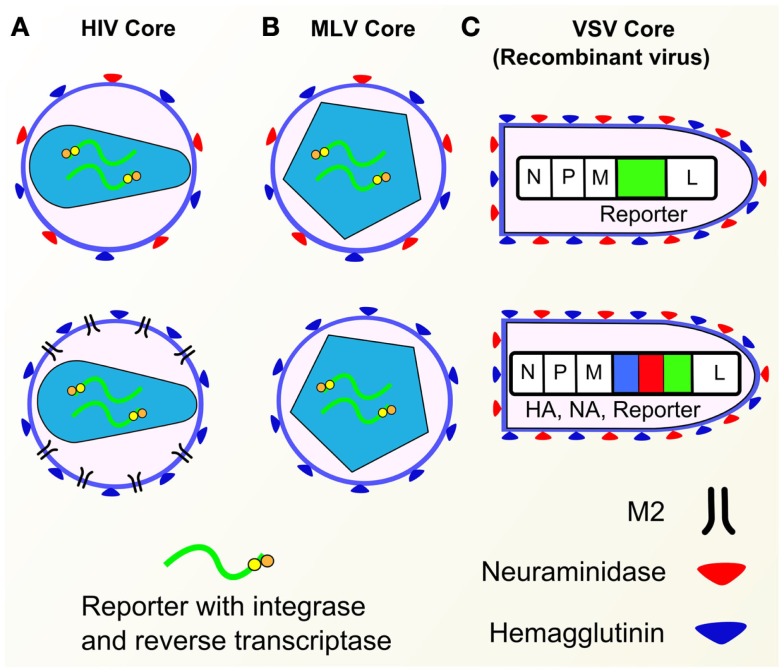
**Pseudotype cores**. **(A)** HIV cores with various envelope glycoproteins (HA, NA, M2). **(B)** MLV cores with HA or HA and NA. **(C)** Recombinant VSV containing GFP gene (top) and HA/NA/GFP genes (bottom). Components of influenza pseudotypes can be varied according to need. Pseudotypes have been produced with HA, NA, and M2 influenza envelope proteins, with a range of core packaging constructs (HIV, MLV, VSV shown) as well as different reporters.

See Figures 4 and 5 for representative drawings of the pseudotype production process and different cores used.

## Production Methods

### Plasmids ratios and amounts

There is considerable variation between studies regarding choice of expression plasmids as particular systems are established within research groups and networks, inherited from previous studies and are often dependent on collaborations or gifts. The most popular system employed involves a multiple plasmid co-transfection approach using separate plasmids for the HA, reporter and retroviral *gag* and *pol* core genes. These genes are cloned into a range of expression plasmids such as pI.18, pcDNA3.1, phCMV, and pCAGGS (43, 74, 103, 104). Kozak consensus sequences are very rarely mentioned and only defined in one study, in which a kozak consensus sequence derived from the pHW2000-N1 (Kan) plasmid was used (3).

Additional plasmids encoding NA and M2 are sometimes used when studying the relevant aspects of influenza infection or pseudotype production, but more rarely in the case of neutralization (93, 105). In one study, a 10- to 30-fold increase in pseudotype production was achieved through expression of M2 using lentiviral cores and a 5-fold increase was achieved when an MLV core was used (29).

Plasmid ratios are crucial to pseudotype production but specific to plasmids used as well as transfection methods. In order to attain the highest quality and yields, optimization is required. Typically, the “core:HA:reporter” plasmid ratio is 1:1:1.5. However, the “HA:NA” ratio ranges from 3:1 to 8:1 and protease gene bearing plasmids (HAT, TMPRSS2) are often present at 50% (or below) the concentration of HA (e.g., 1 μg HA plasmid to 0.5 or 0.25 μg protease plasmid). Calcium phosphate precipitation requires the highest plasmid input, with as much as 25 μg of each plasmid per 100 mm dish used, whereas other methods [Fugene, polyethylenimine (PEI), Lipofectamine] require quantities of between 1 and 5 μg for each plasmid per 100 mm dish (20, 29, 69). Plasmid ratios are differentially affected by the composition of plasmids and therefore the quantities used to produce pseudotypes in the literature are justified based on optimization carried out by particular laboratories (17, 72, 88).

### Producer cells

The producer cell lines used for pseudotype production are predominantly Human Embryonic Kidney 293 cells transformed with the SV40 large T antigen (HEK293T, 293T). These cells are highly susceptible to transfection and make good retroviral packaging cells (106). The clone 17 (HEK293T/17) of this cell line is also extensively used to produce high-titer influenza pseudotypes. Other cell lines used include 293FT cells (Invitrogen) used in the production of VSV–HA–NA pseudotypes (3).

Where mentioned, cell confluency at transfection varies between 60 and 90% with cells subcultured 24 h before transfection (74). Cell monolayers are grown on dishes ranging from 60 to 150 mm with the occasional study using T75 Flasks or multi-well plates (74, 107). Transfections are usually carried out using medium with serum such as fetal bovine serum (FBS) at concentrations of up to 10% (83, 108, 109).

### Transfection reagent/method

The methods studied use the following chemical transfection reagents: Lipofectamine, Lipofectamine 2000 (Thermo Fisher Scientific), Fugene-6, Fugene-HD (Promega), PEI, jetPEI (Polyplus Transfection), or calcium phosphate precipitation. The choice of reagent is based on optimized lab protocol, cost, as well as the cytotoxicity of each reagent depending on requirements of pseudotype production. Of the above reagents, calcium phosphate precipitation and Fugene-6 are the most popular.

Calcium phosphate precipitation is a well-established transfection method of mammalian cells, developed in 1973 by Graham and van der Eb. This method involves mixing a comparatively high amount (5–25 μg) of plasmid DNA with calcium chloride and then adding this mixture slowly to a buffered saline solution. The mixture is incubated at room temperature whereupon a positively charged DNA and calcium phosphate precipitate is formed. The charge allows the precipitate to associate with the negatively charged cell membrane, entering by endocytosis or phagocytosis. The calcium phosphate precipitation process is sensitive to small differences in pH (20, 110, 111).

Polyethylenimine is a polymeric cation, which was first evaluated for its transfection capabilities in 1995. PEI acts at a range of pH values and associates with DNA to produce a complex with an overall positive charge that can then allow interaction with the cell membrane. Entry is by endocytosis and PEI has been shown to aid the delivery of nucleic acids to the cell nucleus of transfected cells. The original report states that PEI is non-cytotoxic at optimal concentration for transfection (112). However, when using PEI for transfection, it is commonplace to change cell culture medium within 24 h of transfection. JetPEI is a manufactured linear form of PEI, which is suited to high-throughput assays (84, 113).

Lipofectamine (or Lipofectamine 2000) are cationic lipids sold by Invitrogen that allow delivery of nucleic acids such as vectors into host cells through the formation of positively charged liposomes. The liposomes containing the pseudotype vectors are then able to fuse with cell membranes due to their positive charge and lipid constitution (114). Lipofectamine is among the most expensive transfection reagents used in influenza pseudotype production. The benefits of using this method are not readily apparent when the cost of the reagent is considered (107, 115).

Fugene-6 and Fugene-HD are cationic lipid complexes, which have low cell cytotoxicity. This allows laboratories to avoid replacing the transfection medium that may allow an increase in final titers of pseudotype when harvested. Fugene-HD has been shown to be more efficient than other transfection reagents (17, 96, 116, 117).

Table 1 shows a list of transfection reagents, their cytotoxicity, cost, and plasmid input required.

**Table 1:** **Transfection reagents, price, cytotoxicity, and plasmid input**.

Transfection reagent	Price	Cytotoxicity	Plasmid input
Lipofectamine 2000	High	–	Low
Fugene-6	High	Low	Low
Fugene-HD	High	Low	Low
Polyethylenimine (PEI)	Low	Low	Low
Jet PEI	Medium	High	Low
Calcium phosphate precipitation	Low	–	High

### Cell washes and medium replenishment

In transfections where cytotoxic reagents are used, medium is replenished 6–24 h post-transfection, with most studies stating that media is typically replenished after overnight incubation (73). Media replacement can also be accompanied by a PBS wash. Where rhabdoviruses are used for pseudotyping, cell lines are washed using PBS 12 h after transfection with influenza surface glycoprotein plasmids. Helper virus is then added and 4 h later the helper virus containing medium is replaced after a further PBS wash step (3).

### Sodium butyrate

Sodium butyrate, a compound that can increase cell proliferation and pseudotype production is used in several studies with the concentrations ranging from 10 μM to 10 mM (18, 28, 57, 68).

### Harvest

Pseudotypes are harvested at various time intervals, typically 48 h post-transfection but sometimes also at 24 or 72 h. The supernatant is taken from the transfected cell monolayer and passed through a 0.45 μM filter to remove cell debris before being stored at −80°C. In many cases, centrifugation at low or high speed is used to concentrate harvested virus (20, 29, 57, 68, 77, 89, 105, 115, 118, 119).

One study has demonstrated that influenza pseudotypes are stable after five freeze–thaw cycles, retaining over 80% infectivity. Keeping pseudotype supernatant at −20°C for 6 months had a similar effect. However, storage at −4 or 20°C led to a reduction in infectivity of 50% in both cases (120). In environments lacking reliable refrigeration facilities, pseudotypes can be lyophilized and stored at a range of increased temperatures and humidity, maintaining viability and concentrations adequate for use in neutralization assays (121).

**Figure 6:**
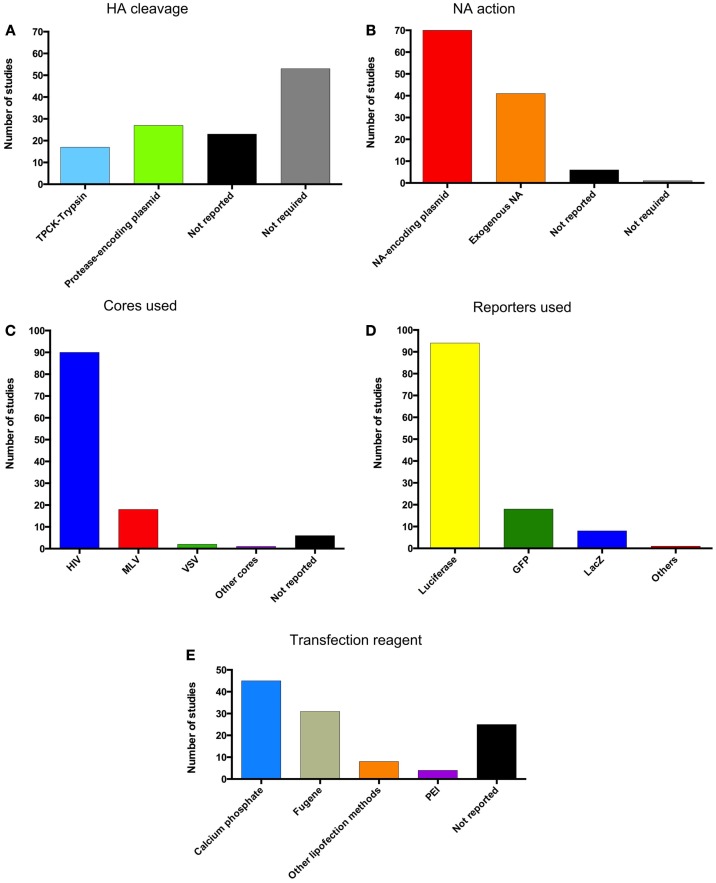
**Pseudotype production methods**. Graphical representation of the methods used for pseudotype production in the literature cited in this review. **(A)** Method of HA cleavage used. **(B)** Method of NA action used. **(C)** Pseudotype cores used. **(D)** Reporters incorporated into pseudotypes. **(E)** Transfection reagents for the production of pseudotypes.

See Figure 6 for a detailed depiction of methods used for production based on all pseudotype employing articles cited in this review.

## Transduction

### Titration

As previously mentioned, with luciferase reporter pseudotypes RLU readings derived from titrations can be used as a secondary measure of pseudotype concentration within a sample. However, RLU readings are dependent on many variables surrounding the cells and the particular luminometer used.

Pseudotypes are titrated by 2-fold serially diluting 100 μl of harvested supernatant in a 96-well plate. After an incubation of 48 or 72 h, RLU can be measured by lysing the transduced cells and adding luciferin (luciferase substrate). This can then be used to calculate the RLU per well and the RLU/ml of the original sample.

Reverse transcriptase quantitative PCR (qRT-PCR) has also been employed in order to estimate transfected gene copies as well as mRNA copies in cells. This method is often used in conjunction with others described in this section in order to have comparative measurements of pseudotype quantity (55, 109, 122).

In many studies, pseudotype input is normalized via enzyme-linked immunosorbent assay (ELISA) detection of the principal component of the HIV core, p24 (16, 17, 28, 54, 59, 88, 89, 92, 95, 103, 109, 113, 115, 123, 124). However, as core budding is independent of surface HA, this method will detect cores lacking envelope glycoproteins as well as cores belonging to transduction competent pseudotypes. Pseudotype HA has also been detected using ELISA and used to normalize pseudotype input (82, 98, 125).

Quantification through hemagglutination assay has also been used frequently (28, 55, 58, 82, 84, 95, 98, 101, 122, 126–128).

Western blotting is used in some cases to determine the amount of glycoprotein or HIV p24 in a pseudotype sample (59, 72, 109). It is also used in a wider range of studies to ascertain glycoprotein or HIV p24 expression (17, 28, 55, 109, 122).

### Cell input

The vast majority of studies involving neutralization assays titrate and transduce in 96-well plates with 1 × 10^4^ cells (HEK293, HEK293T/17, or MDCK) per well. However, the amount of cells can range from 5 × 10^3^ to 1 × 10^5^. In some instances, 293A and MDCK-London cells are also used, whereas BHK-21 cells are frequently used for VSV-based pseudotype infection due to their comparative susceptibility (65, 73, 93, 95, 129–132). Specialized cells overexpressing α2,6-linked sialic acid (MDCK-SIAT) have also been used and compared to parental cells in the presence of soluble HA (77).

In one case, transduction was carried out in 96-well transparent culture plates, before lysates were then transferred to 96-well luminometer plates for analysis (69). The importance of pseudotype input in batch to batch variation is highlighted in Garcia et al. (133), the study suggests that an RLU of at least 1 × 10^5^ per well should be used to ensure that antibody titer is independent of pseudotype input.

### Substrates

Steady-Glo or Bright-Glo (Promega) are the most common sources of luciferin. While expensive, these two substrates also serve a secondary purpose of lysing cells and releasing any expressed luciferase enzyme.

### Equipment: 96-well plates and luminometers

There is some disparity in the recording of equipment used in the articles studied for this review. Without this required information, reproduction of each study is hampered by these further variables relating to plate reading. Information relating to the color and manufacturer of 96-well plates is very important in the quantification of viable pseudotypes in order to prevent introduction of further variables between laboratories. While logistically difficult, the standardization of neutralization assay equipment across laboratories studying influenza would bring benefits to the interpretation of research data. Standardization of plate reading equipment is also required in order to ensure comparable data are obtained from different machines when reading the same experiment.

### High-throughput approaches

A high-throughput approach has been used to evaluate antiviral compound effects on pseudotype transduction, testing a wide range of unique compounds in a single assay performed with 96- or 384-well plates (115, 134).

### Increased transduction efficiency

Polybrene (hexamethrine bromide) and polyfect (Qiagen) are used in several studies in order to increase transduction efficiency (17, 29, 58, 68, 76, 79, 80, 98, 135). 1 μg/ml, 8 μg/ml, or 16 mg/ml of polybrene is added to virus or virus/antibody mixes before the addition of cells in titration and neutralization assays or during incubation.

In two studies, spinoculation was used to increase transduction rate. To achieve the increased transduction rates, the pseudotypes and cells were centrifuged at 1250 rpm for 2 h or 3000 rpm for 1 h (3, 92).

## Pseudotype Neutralization Assays

### Protocol

**Figure 7:**
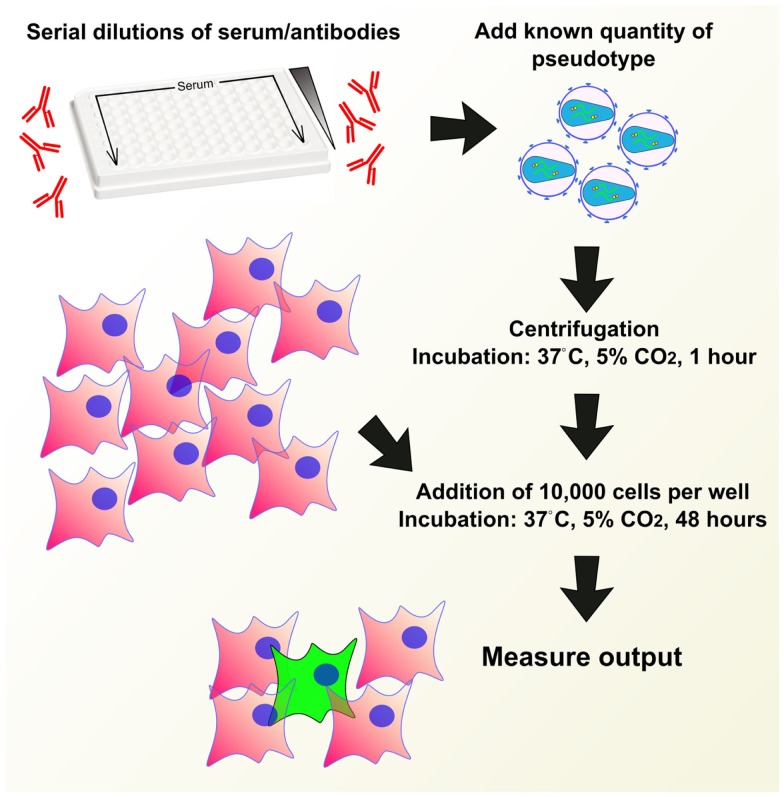
**Example of a pseudotype neutralization assay (pMN)**. Serum or antibodies are serially diluted across a 96-well plate, a known quantity of pseudotype is added and the plate is centrifuged and incubated to allow antibody binding. A set quantity of cells are added and plates are incubated for 48 h. Output is measured in a manner depending on reporter used.

Pseudotype neutralization assays (pMN) are usually carried out in 96-well white plates. A measured amount of antibody in medium is serially diluted across the plate and incubated with a set amount of quantified virus in medium, usually at a 1:1 virus:antibody ratio. Incubation is carried out at between 20 and 37°C for between 30 min to 2 h (43, 82, 96, 123, 133, 136). About 1 × 10^4^ target cells are then added to each well, subsequently the plate is left to incubate at 37°C in 5% CO_2_ for 48 or 72 h. A cell-only control as well as known positive and negative sera standards should be used as benchmarks for the neutralization assay (95, 113). See Figure 7 for a depiction of the pMN assay.

### Pseudotype input

The quantities of pseudotype used in neutralization assays, which were normalized based on p24 ELISA ranged from 6.25 to 50 ng/ml (17, 95). RLU or RLA values of between 1 × 10^4^ and 1 × 10^6^ per well were used (in a 96-well plate), sometimes in conjunction or normalized with p24 or qPCR methods (89, 121, 127). Estimates of copy number per set volume of original viral supernatant can also be used. It is important to note that RLU based values are affected by the make-up of the plasmid bearing the HA gene, as well as a multitude of factors such as the luminometer, which is used to measure transduction.

### Serum/antibody dilutions and start points

Antibody input varies depending on availability, especially when taking into account the possibility of repeats and replicates. Antibodies are primarily diluted 2-fold in Dulbecco’s modified eagle medium (DMEM), with or without FBS, across a 96-well plate, with the occasional three, four, or 5-fold dilution experiment (3, 18, 58, 137, 138). Where mentioned, starting antibody concentration ranged between 1:4 and 1:40.

### Incubation times and time periods

When stated, serum complement inactivation varies from 30 min to 1 h at 56°C (68, 133). Pseudotype-antibody incubation times are generally consistent between studies, at 37°C for 1 h. Transduction times vary in 24 h increments at 24, 48, and 72 h, after which output is measured.

### Controls

Positive sera or specific commercial antibodies are required as positive controls, which can be compared to tested sera and used to normalize between assays (see Approaches Toward Validation and Standardization). Reference sera from the National Institute for Biological Standards and Control (NIBSC), Office International des Epizooties (OIE), Animal and Plant Health Agency (APHA, previously AHVLA), and US Food and Drug Administration (FDA) are regularly used (88, 89, 127, 139, 140).

### Neutralizing antibody titer determination

Antibody effect is displayed using one of many inhibitory concentrations (IC_50_, IC_80_, IC_90_, and IC_95_). The numerical value relates to the percentage point each particular study is calculating. For example, the IC_50_ value can represent the concentration of an antibody that reduces RLU reading by 50%, when compared to 100 and 0% transduction controls (48, 68, 141, 142). These controls are essential to the calculation. About 100% inhibition can be benchmarked by a cell-only control and 0% by incubation of cells and virus in the absence of sera.

### Hemagglutination-inhibition assay

Hemagglutination-inhibition assay (HI) assays using pseudotypes utilize the same procedures as with wild type virus. A quantified amount of viral sample (as determined by hemagglutination assay) in phosphate buffered saline is added to serially diluted sera in a 96-well plate, to which 50 μl of a 0.5–1% chicken/turkey red blood cell suspension is added. After 30 min to 1 h, the HI plates are scored for agglutination. Pseudotype input is adjusted according to WHO guidelines at four hemagglutination units and sera is treated with receptor destroying enzyme to inactivate non-specific inhibition of agglutination (37, 98).

### Post-attachment assay

The post-attachment neutralization assay is used to identify antibodies that neutralize HA after it has bound to sialic acid. Oh et al. (143) modified the post-attachment assays, originally developed by Edwards and Dimmock (144), to allow wild type influenza virus to be replaced by influenza pseudotype particles.

In this assay, pseudotype particles are incubated at 4°C with cells to enable the synchronization of the attachment of virus to sialic acid on the cell surface and to block viral endocytosis. A diluted serum is then added, and following another 4°C incubation, plates are transferred to 37°C to permit transduction (143). Transduction is then measured using the same approach as that taken in a neutralization assay.

Antibodies detected by this assay have neutralizing activity via their ability to impede the endocytosis step and subsequent HA conformational changes necessary for virus–endosome fusion (143, 144). Antibodies that have neutralizing activity through impeding viral attachment will produce negative results in this assay. The post-attachment assay is useful for evaluating the neutralizing capacity of stalk-directed antibodies that do not inhibit viral attachment (143, 145).

### Cross reactivity

The issue of cross-reactive sera has been raised previously in traditional serological assays, serum samples produced by injection of wild-type virus into mice have been shown to lead to the presence of interfering antibodies directed toward NA or M2 epitopes (146, 147). It is expected that pMN will suffer from the same problems of cross-reactivity, an important issue, which must be addressed in the future in order to strengthen the usefulness of this assay as a competitor to the current gold standards.

## Reproducibility

Reproducibility is a major issue in the field of serology. Serum samples are often finite, leading to an inability to reproduce experiments or results in the same context as they were originally published. However, by standardizing methods for production, titration, and neutralization and the use of common reference standards it is possible to minimize variation between experiments and research groups.

## Correlation with Other Serological Assays

Comparisons have been made between pMN assays and traditional serological assays with mixed results. Several articles report increases of between 31.9 and 200% in human antibody titers in comparison to microneutralization (MN) based results (148, 149). Buchy et al. (148) show a correlation between H5 pseudotypes and MN (spearman 0.79, *p* < 0.001), which is also seen in Du et al. (69) and Wang et al. (89), the latter presenting *r*^2^ values of 0.9802 for A/Vietnam/1203/2004, 0.8193 for A/Anhui/1/2005, and 0.5244 for A/turkey/Turkey/1/2005 strains.

Alberini et al. (137) compared pMN assays to hemagglutination-inhibition (HI), single radial hemolysis (SRH), and MN assays using 226 different human serum samples. The Pearson correlation test produced significant correlation (*p* < 0.001) between the antibody titers calculated from each assay. The correlation coefficients between pMN and HI, SRH, and MN assays were 0.73, 0.70, and 0.78, respectively. Furthermore, the correlation between H5 MN and H5 pMN allowed the establishment of a threshold from which pMN titers could be based. pMN data were then analyzed based on the threshold, which showed protective titers in patients of 38–43, 54, and 79% after adjuvanted vaccination, second dose and booster, respectively (137).

Qiu et al. (81) show a range of correlations between HI and pMN using different HA subtypes. A/Moscow/10/1999 (H3N2) correlates well (*r* = 0.8454, *p* < 0.0001), A/Brisbane/59/2007 (H1N1), and A/Japan/305/57 (H2N2) poorly (*r* = 0.1171, *p* = 0.7472 and *r* = 0.1171, *p* = 7472) whereas A/Vietnam/1203/2004 (H5N1) correlates (*r* = 0.7921, *p* = 0.0029). In an additional study, HI and pMN (IC_50_) correlate well in Qiu et al. (107) in the case of A/Shanghai/4664T/2013 (H7N9) (spearman *r* = 0.88, *p* < 0.0001) as well as in Whittle et al. (126) (*r*^2^ = 0.6491, *p* < 0.0001).

A significant correlation of 65% (*p* = 0.002, *r* = 0.65) has also been reported between SRH and pMN using equine influenza pseudotypes and sera and another study showed the relationship between RLU and HA content (78, 119).

## Approaches toward Validation and Standardization

Approaches toward the standardization of pMN should follow the procedure that was required for MN standardization. Standardization of MN in general has focused on the use of pooled serum samples as reference standards. A/California/7/2009 (pandemic H1N1, pdm) standard was established by the WHO in 2010 with an assignment of potency of 13,000 IU/ml. A second pooled sera reference standard for H5N1 exists and has successfully been used in a number of studies (89, 137, 150). A cut off value for positive and negative H5N1 neutralizing sera exists for this set of H5N1 reference standards (137).

## Chimeric Hemagglutinin and Stalk-Directed Antibodies

There has been considerable research into the stalk region of HA in relation to vaccine design and immunity to influenza. Various stalk-directed monoclonal antibodies (mAB) such as CR6261 have been characterized, opening up the potential use of chimeric HA to test for the presence of similar antibodies in serum samples (151, 152).

Stalk-directed antibodies were first identified in 1994 when the cross-reactive C179 mouse monoclonal antibody was identified and found to inhibit fusion of several HA subtypes (153). Since then many studies have focused on stalk-directed antibodies and their neutralization of multiple diverse subtypes of influenza (145, 152, 154–156). However, this range of heterosubtypic immunity is dependent on the characteristics of the epitope of each antibody tested, which will influence which subtypes, clades and whether they neutralize group 1 or 2 influenza.

The stalk region of HA is more conserved than the variable globular head to which the vast majority of neutralizing antibodies are directed. While residues in the head mediate attachment of the virus to target cells by binding to sialic acid, the fusion peptide in the stalk of HA is just as crucial to the HA function (157, 158). In order to test for neutralizing stalk antibodies, studies have employed a variety of chimeric HA constructs bearing stalks and heads from different subtypes. The concept behind this revolves around the use of HA heads that are largely unreactive to the antibodies used in the assay. Utilizing this approach, a neutralizing response can be detected in the absence of head-directed neutralization.

Several hybrids have been constructed and pseudotyped using HIV cores, these are generally constructed through PCR amplification and incorporation of complementary restriction sites, allowing ligation of different segments of HA genes. A wider variety has been used in reverse genetics approaches toward development of wild type virus bearing chimeric HAs (159–161). These chimeric HA are promising candidates for the testing of “universal” vaccines.

**Figure 8:**
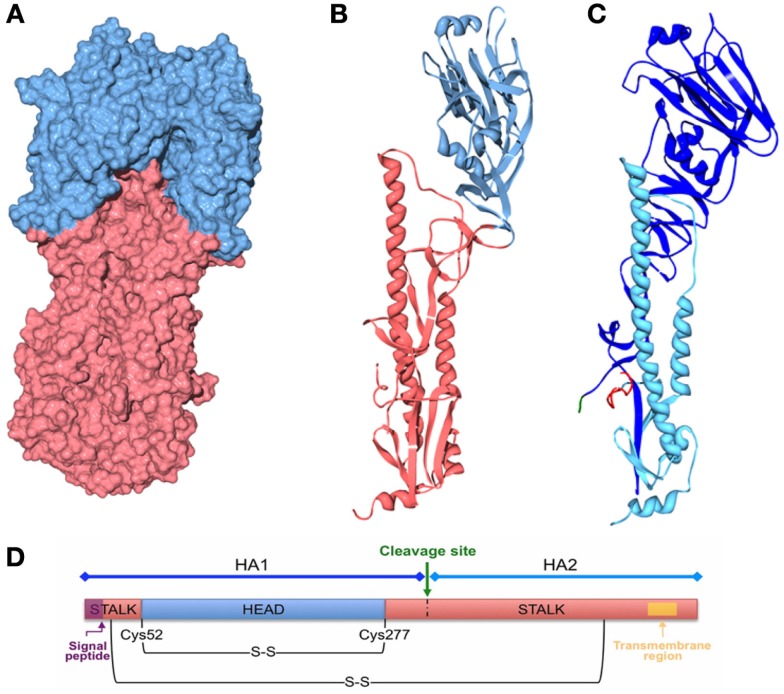
**Computer models of chimeric HA**. Three-dimensional structures were generated with Swiss PDB viewer and POV-Ray 3.7 using the structure of the recombinant virus A/Hong Kong/1/1968 X-31 H3 [PDB ID: 2VIU (162)]. The signal peptide is not present in the HA. The transmembrane region is not resolved by X-ray crystallography. **(A)** Three-dimensional structure of the influenza HA trimer, showing the HA surface of the head (blue) and stalk (red) regions. **(B)** Three-dimensional ribbon structure of the influenza HA monomer showing the head (blue) and stalk (red) regions. **(C)** Three-dimensional ribbon structure of the influenza HA monomer showing HA1 (blue) and HA2 (light blue) subunits, the cleavage site and the fusion peptide are also shown in green and red, respectively. **(D)** Schematic of the HA polypeptide.

Table 2 displays the regions and subtypes used in the construction of chimeric hemagglutinins. Figure 8 is a visualization of chimeric HA construction in the form of a computer model.

**Table 2:** **Examples of chimeric hemagglutinins originating from divergent subtypes and used for pseudotype production**.

Reference	Head	Stalk
Hai et al. (58)	H5 A/Vietnam/1203/2004	H1 A/Puerto Rico/8/1934
	H1 A/California/04/2009	H1 A/Puerto Rico/8/1934
	H7 A/mallard/Alberta/24/2001	H3 A/Perth/16/2009
	H5 A/Vietnam/1203/2004	H3 A/Perth/16/2009
Pica et al. (80)	H6 A/mallard/Sweden/86/2002	H1 A/Puerto Rico/8/1934
	H9 A/guinea fowl/Hong Kong/WF10/1999	H1 A/Puerto Rico/8/1934

	**HA1**	**HA2**

Wang et al. (72)	A/Brisbane/59/2007	A/New Caledonia/20/1999
	A/New Caledonia/20/1999	A/Brisbane/59/2007

## Future of Influenza Pseudotypes

Pseudotype neutralization assay offers the safety of using pseudotypes and the sensitivity of the MN assay. Further validation and standardization of the assay are required but once established, the assay should offer a robust and sensitive means of interrogating influenza vaccine trials for head and stalk-targeting antibodies. The production of vaccines that elicit stalk-targeting antibodies may in time lead to a universal vaccine, preventing 250,000–500,000 deaths from seasonal influenza and the emergence of pandemic strains, most recently the H1N1 2009 pdm, which caused an estimated 284,500 deaths (163). pMN currently offers the opportunity to batch test vaccines or commercialized antibodies in the absence of standardization.

Furthermore, the ability of the pMN assay to include chimeric HA, and also NA and M2 allows the pMN to be used to explain the pathogenicity of seasonal and pandemic influenza strains and perhaps elucidate the antigenic evolution of influenza further.

## Other Uses of Pseudotyping Influenza

### Gene therapy and vaccines

As the field of gene therapy progresses, influenza pseudotyping will benefit from the design of even safer and more effective vectors. As more sophisticated systems are developed they may become more easily standardized and comparable to wild type virus.

One aspect of gene therapy that may benefit the field of influenza is the use of viral entry proteins to target delivery of nucleic acids into specific cells, as vaccines or delivery systems. One delivery system study used influenza pseudotypes to transduce the respiratory epithelial cells of mice after nasal administration with promising results indicating that the method could be used in the treatment of cystic fibrosis (118). A similar study presented the rescue of ciliary function using influenza pseudotypes containing therapeutic cDNA (164).

Pseudotype-based influenza gene delivery vaccines are also becoming more widespread, with several candidates already cited in this review. Baculovirus pseudotyped with VSV-G has been used successfully to express HA in mammalian cells and provided an efficacious vaccine when tested in chickens and mice (165). Originally a popular vector for transgene expression in insect cells, baculovirus has been shown to be a useful tool for vaccine production in mammalian cells (166). In Wu et al. (165), delivery was achieved through VSV-G incorporation into baculovirus under the effect of the polyhedron promoter and HA under the effect of the CMV promoter in order to achieve expression and subsequent infection of mammalian cells. This is an interesting gene delivery system, which could be used as a method for the introduction of pseudotype genes into cells through a VSV-G bearing baculovirus in lieu of cytotoxic transfection reagents.

A further pseudotype vaccine has been developed which contains a modified HA gene, allowing expression in transduced cells but lacking the viral RNA sequences required for replication. This approach yields a particle bearing the desired glycoproteins, in this case A/Puerto Rico/8/1934 (H1) that consequently induces a robust T-cell response when given to mice via inhalation. Reduction in the severity of symptoms was also seen in mice infected with a different subtype: H3N2, A-X31 (71). While these approaches demonstrate the flexibility of the pseudotype platform, other more established methods including adenovirus or modified vaccinia viruses (e.g., modified vaccinia Ankara) may present a more attractive option for the delivery of influenza genes, and have been reviewed in great depth (167).

Pseudotypes used as immunogens, such as those bearing H5 have been tested in mice as a candidate vaccine, eliciting high levels of anti-HA antibodies as determined by HI. Mice that were vaccinated survived despite weight loss of approximately 12.8–21.1% whereas the non-vaccinated group lost approximately 25.5–26.2% of their bodyweight and perished 6 days after H5N1 virus challenge (20). A similar approach is taken by Szécsi et al. (168) in the production of H5 and H7 pseudotyped virus-like particles as immunogens tested in mice.

Influenza pseudotypes could also be used in vaccine design through the use of integrase defective lentiviral vector technology. Defective lentiviral vector technology allows transduction of target cells through maintenance of an episomal reporter construct without integration into the genome. This approach may bring benefits by reducing the chance of interrupting host genes and the eventual dilution of the delivered gene over time (169, 170).

**Figure 9:**
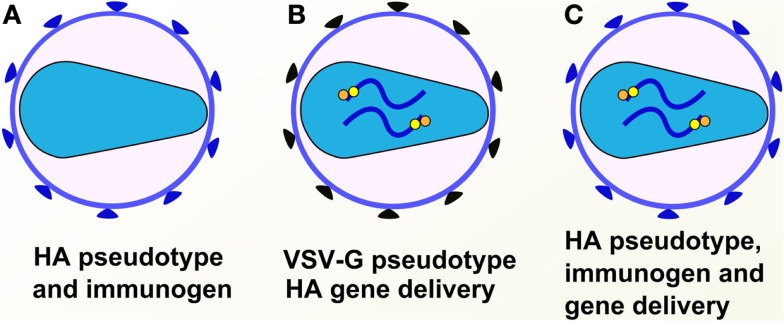
**Pseudotypes used for gene delivery or as immunogens**. Pseudotypes can be employed as immunogens bearing the antigen of choice or as delivery systems for genes of choice. **(A)** HA-based pseudotype/virus-like particle immunogen. **(B)** VSV-G pseudotype delivery system for HA gene. **(C)** HA pseudotype delivery system for HA gene.

See Figure 9 for a depiction of the various pseudotype-based vaccines and immunogens.

## Recommended Consensus Protocol from Synthesis of Published Articles

### Production protocol

A HEK293T cell monolayer of 60–90% confluence should be transfected using Fugene-6 or calcium phosphate precipitation in medium containing 10% FBS. Plasmid ratios should be optimized based on the plasmids used. The use of second generation HIV packaging constructs is recommended. An NA encoding plasmid can be used or exogenous NA can be added at 24 h post-transfection to induce release of pseudotypes. The supernatant should be harvested at 48 h post-transfection and filtered through a 0.45 μm filter. Filtered supernatant should be kept at −80°C in single use aliquots if long-term storage is required.

### Titration protocol

Titration should be carried out using luciferase-based transduction in 96-well white plates, by p24 ELISA or other methods of quantification. Quantification of pseudotype particles using luciferase-based transduction involves the 2-fold serial dilution of 100 μl of pseudotype in 10% FBS medium. About 1 × 10^4^ cells are then added in a 50 μl volume and the resulting solution is incubated for 48 h. After the 48-h incubation period luciferase substrate is added to each well and RLU values are read. Cell only, ΔEnv and VSV-G bearing pseudotypes can be used as negative and positive controls.

### Pseudotype-based neutralization protocol

Serum samples are serially diluted across a 96-well plate in 50 μl of media. Pseudotype virus should be added in a 50 μl volume at a concentration of 1 × 10^6^ RLU. After 1 h incubation at 37°C, 1 × 10^4^ HEK293T or MDCK cells should be added in a 50 μl volume. The plate is then incubated at 37°C for 48 h before luciferase substrate is added to each well, after which RLU values are read. Standards should ideally be used in the form of neutralizing antibodies or pooled serum samples.

## Conflict of Interest Statement

The authors declare that the research was conducted in the absence of any commercial or financial relationships that could be construed as a potential conflict of interest.
